# Is It Easy to Be Urban? Convergent Success in Urban Habitats among Lineages of a Widespread Native Ant

**DOI:** 10.1371/journal.pone.0009194

**Published:** 2010-02-12

**Authors:** Sean B. Menke, Warren Booth, Robert R. Dunn, Coby Schal, Edward L. Vargo, Jules Silverman

**Affiliations:** 1 Department of Entomology, North Carolina State University, Raleigh, North Carolina, United States of America; 2 Department of Biology, North Carolina State University, Raleigh, North Carolina, United States of America; 3 W. M. Keck Center for Behavioral Biology, North Carolina State University, Raleigh, North Carolina, United States of America; Field Museum of Natural History, United States of America

## Abstract

The most rapidly expanding habitat globally is the urban habitat, yet the origin and life histories of the populations of native species that inhabit this habitat remain poorly understood. We use DNA barcoding of the COI gene in the widespread native pest ant *Tapinoma sessile* to test two hypotheses regarding the origin of urban populations and traits associated with their success. First, we determine if urban samples of *T. sessile* have a single origin from natural populations by looking at patterns of haplotype clustering from across their range. Second, we examine whether polygynous colony structure – a trait associated with invasion success – is correlated with urban environments, by studying the lineage dependence of colony structure. Our phylogenetic analysis of 49 samples identified four well supported geographic clades. Within clades, Kimura-2 parameter pairwise genetic distances revealed <2.3% variation; however, between clade genetic distances were 7.5–10.0%, suggesting the possibility of the presence of cryptic species. Our results indicate that *T. sessile* has successfully colonized urban environments multiple times. Additionally, polygynous colony structure is a highly plastic trait across habitat, clade, and haplotype. In short, *T. sessile* has colonized urban habitats repeatedly and appears to do so using life history strategies already present in more natural populations. Whether similar results hold for other species found in urban habitats has scarcely begun to be considered.

## Introduction

Unlike most habitats, urban and managed environments are growing in area in nearly all geographic regions [1]. In the language of global change, these expanding urban environments are “non-analogue”, which is to say they do not have a direct analogue in contemporary natural conditions [2], [3]. These changes in global environments have favored a narrow subset of species [4]–[7], including invasive species, introduced beyond their native range [6], [8], [9], but also species native to the regions [7], [10]–[13]. Yet, while a large body of work has focused on the history and traits of invasive species [14]–[16], only a small handful of studies have considered the traits associated with and origin of urban populations of native species [7]. Put simply, how do urban populations of native species arise?

The urban habitat is one of the fastest growing habitats globally, and the habitat in which most humans now live [1]. As such, the species with the most interactions with humans are and will increasingly be those in urban environments [8], [12]. The study of the evolutionary origins of such species and their traits is a new and growing field [7]. The differences in life histories of species in urban environments relative to in their native habitats are diverse, but include increased geographic distributions, decreased home ranges, higher densities, changes in foraging, increased reproductive output, and other life history traits [7], [11], [14], [16]–[19]. At least some of the traits that seem to be favored in urban environments are also those that are favored in invasive species and more generally disturbance specialists, such as short generation times, rapid reproduction, good dispersal, and plastic behaviors and life history traits [4], [15], [20]. In invasive species, at least some of the traits present in the invaded range but absent in the native range represent microevolutionary responses to novel environments. Whether the same is true for native species in the urban environment is unclear.

**Figure 1:**
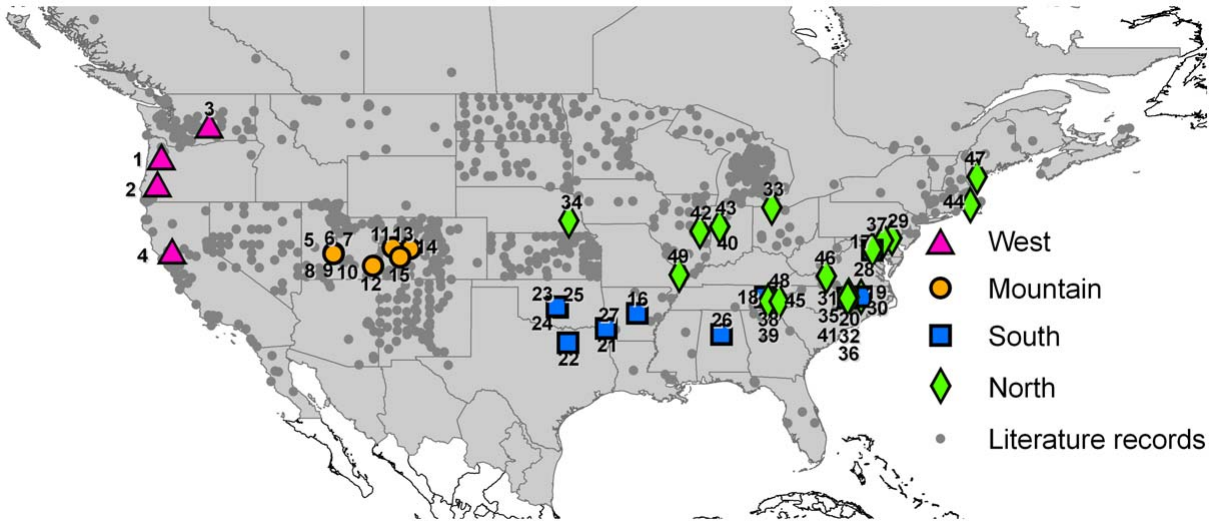
Distribution of *Tapinoma sessile* North America. Museum and literature collection records (small gray circles), and the 49 localities represented in this study (west – purple triangles; mountain – orange circles; south – blue squares; north – green diamonds).

Globally, the family of ants (Formicidae) is one of the most prominent groups of organisms, and includes many species that perform important ecological services relevant to humans and the ecosystems on which humans depend [21]. Many ant species that perform these services persist in urban environments [22]. In addition, ants may perform services in urban environments that are not necessarily valuable elsewhere, such as the removal of food, waste and other items. However, most urbanized environments are also accumulating pest species (species that pose problems for humans), including invasive and native species some of which are of economic and public health concern, species it might be said, that perform disservices [23]–[26]. While the native species that inhabit urban environments have received some attention in the pest control literature [23], [24], their origins and basic biology remain essentially unexplored. For example, *Tapinoma sessile*, the odorous house ant, is the most widespread native ant in the United States, occurring in most habitat types between the eastern and western coasts of northern Mexico to southern Canada from sea level to 4,000 m (Fig. 1, unpublished data, http://www.antmacroecology.org/projects.html). It has also been a common structural pest species for over 100 years [23], [27], both inside and outside of buildings [23], [28] and it has been reported that it is rapidly expanding its range in urban environments [29]. Yet only a few studies, all taking place in urban areas [27], [30]–[32], have explicitly studied its basic biology, and only a few more studies [33]–[37] have mentioned anything about the biology of this species in natural habitats. Due in part to a lack of knowledge on the basic biology of this species in different habitats or different parts of its range, little is known about how, when, and why it has been successful, particularly in urban environments.

Although it has not received much study, reports to date suggest that *T. sessile* possesses many features within the suite of traits referred to as the “invasive syndrome” [14], suggesting that such a syndrome may promote success both in non-native habitats but also in non-analogue habitats such as cities. Traits implicated in this syndrome include large colonies, colonies with multiple queens that mate within the nest, and colonies that often occupy many interconnected nests with little to no territorial aggression among them [25]. Fellers [36] and Milford [33] reported that colonies of the odorous house ant were small, occupying single nests, and acted as a subdominant species in natural habitats. In contrast, studies of pest populations in urban habitats have reported that the odorous house ant forms large polydomous populations that act as behaviorally dominant ants [27], [30], [31]. But it remains uncertain whether these invasive traits are actually found only in urban populations of *T. sessile*. Moreover, it is unknown whether there was a single colonization of the urban environment by *T. sessile*, with the subsequent evolution of the “invasive syndrome” traits, or instead, independent biogeographic shifts from native into urban environments. The observation that few native species have become pests in disturbed habitats suggests that the traits necessary for such a lifestyle are uncommon, and that they neither readily evolve nor are very plastic, whether for *T. sessile* or any other ant species. On the other hand, the wide geographic range of *T. sessile* and its occurrence in diverse habitats represent characteristics of a generalist species that can obviously plastically adapt to numerous different environments and may provide important insight about the origin of urban populations.

We chose to address two related hypotheses regarding the phylogeography of *T. sessile*. First we studied whether all urban samples of *T. sessile* have a single common origin from one or more natural populations, suggesting a single successful invasion of a novel environment and subsequent human transport. An alternative hypothesis is that urban populations descend from local natural populations, suggesting repeated successful invasions of a novel environment. We assessed these hypotheses using a phylogeographic approach to determine whether urban samples across the range of the species in the United States are genetically similar to nearby samples found in native habitats. Second, we looked at whether polygynous colony structure, a colony-level trait thought to be linked with success in invading novel habitats, is consistently associated with the transition to urban lifestyle, or alternatively, if it is also common in natural habitats. We assessed this by studying the lineage dependence of colony structure.

## Methods

### Specimen Collection

Forty-nine collections of *T. sessile* were made from 47 localities (Fig. 1, Appendix S1) throughout the continental United States. Specimens were collected by the authors or provided by other biologists and pest management professionals. The habitat at the collection point was divided into two broad categories: urban and natural. At 18 of the collections it was possible to determine if the nest site was monogynous or polygynous by collecting either the entire nest or by collecting numerous queens from the same nest. We considered all multi-queen colonies to be polygynous, but we were not able to ascertain if all queens within in a nest were reproductively viable or contributing brood. In order to determine if colony collections from a nest site represent a complete colony or just a colony fragment from a satellite nest in a polydomous colony, worker aggression assays were used. Single workers from the collected colony were introduced to the nest entrances of adjacent colonies, within a 10 meter (m) radius, that were identified by baiting and visual surveys. If these workers were treated aggressively and killed, we inferred that our collections represented a complete colony, whereas if the workers were accepted at the new nest entrance then the nest collection represented part of a larger polydomous colony. Based upon laboratory trials worker introductions to a different colony always result in worker mortality. Voucher specimens were deposited at the Bohart Museum, Department of Entomology, University of California at Davis (UCD) (Appendix S1).

### Molecular Techniques

Total genomic DNA was extracted from 68 whole individual ants represnting 49 nests of *T. sessile* and 1 nest of *T. erraticum* (see Appendix S1) using DNeasy Tissue extraction kit (QIAGEN, Valencia, CA). Cytochrome oxidase I (COI) gene sequences, commonly used in “DNA barcoding”, were amplified using primers LepF1 (5′-ATTCAACCAATCATAAAGATATTGG-3′) and LepR1 (5′-TAAACTTCTGGATGTCCAAAAAATCA-3′) [38], [39], which targeted a 658-bp fragment. Polymerase chain reactions (PCRs) were performed in 30 µl volumes containing: 1X PCR buffer, 2 mM MgCl_2_, 100 µM dNTPs, 3 pM of each primer, 0.5 U *Taq* DNA polymerase (Bioline, Taunton, MA), ∼50 ng DNA template, and ddH_2_O to 30 µl. PCR cycling conditions were comprised of an initial denaturation stage of 1 min at 94°C, followed by 4 cycles each consisting of 1 min at 94°C, 1.5 min at 45°C, and 1.5 min at 72°C. This was followed by 34 cycles each consisting of 1 min at 94°C, 1.5 min at 45°C, and 1 min at 72°C, carried out using a PTC-200 thermal cycler (MJ Research Inc.). All 68 DNA extracts amplified under these conditions. PCR products were visualized on a 2.5% agarose gel to confirm samples contained a single band, and they were subsequently purified using QIAquick PCR purification kit (QIAGEN, Valencia, CA) and bidirectionally sequenced following the methodology outlined in Copren et al. [40]. Sequence alignments were performed using the Vector NTI Advance 10 program (Invitrogen, Carlsbad, CA).

### Phylogenetic Analysis

Sequence alignment was performed using the Vector NTI (v10) software (Invitrogen). Phylogenetic relationships were initially examined using Molecular Evolutionary Genetics Analysis (MEGA), version 4 [41]. Four methods were employed, Neighbor-joining (NJ), UPGMA, Maximum parsimony, and Minimum evolution. Under these methods all characters were weighted equally and bootstrap replicates set to 10,000. Subsequently, hierarchical likelihood ratio tests for DNA substitution at the COI gene were performed using MrModeltest version 2.3 [42] and Bayesian analysis performed using MrBayes version 3.1.3 [43]. Two independent MCMC analyses, each with one cold and three hot chains, were run for 10^7^ generations. Burn-in was set to discard the first 1000 of 10,000 sampled trees. Convergence of chains to a stationary distribution was assessed by inspection using the computer program Tracer v1.5, and confirmed by obtaining values of the average standard deviation of split likelihood frequencies less than 0.01 between the two runs. *Tapinoma erraticum* was used as an out-group. Sequence divergence within and between clades was calculated using Kimura-2 parameter pairwise genetic distance using MEGA version 4.

The evolutionary relationship between mtDNA sequences was also calculated through the construction of minimum spanning networks using the program TCS 1.21 [44] which follows the method outlined by Templeton *et al*. [45]. Given the sparse collection for some regions we felt that our data set was not suitable to infer significance of phylogeographic patterns observed using tests such as Nested Clade Analyses. Networks found to be unconnected at the 90% probability of parsimony were linked by decreasing the connection probability to a minimum of 60%.

Analysis of molecular variance (AMOVA) was used to assess genetic structure hierarchically based on geographic clustering (following identification by phylogenetic tree and minimum spanning network construction) and habitat association. Calculations were performed using the Arlequin 3.11 software [46].

## Results

### Ecological Data

Of the 49 *T. sessile* nest collections that were processed, colony structure and habitat varied widely, from an acorn-nesting monogynous colony with about 50 workers in a deciduous forest (sample 43 – north clade), to a polygynous colony containing two queens and approximately 250 workers nesting under a rock in a high elevation meadow (sample 14 – mountain clade), to a very large colony with millions of workers and thousands of queens nesting in and around several buildings on a college campus (sample 36 – north clade). Colony structure was recorded in 18 of the collections based on number of queens collected at one nest site. Twenty-three collections were made in natural environments, most of which were stray workers collected by entomologists without any information on colony structure. Twenty-six collections were made in urban environments, most provided as stray workers by pest management professionals from infestations in homes. For the six monogynous colonies sampled, the entire colony was hand-collected and workers introduced to the nearest surrounding nests were all killed demonstrating colony differentiation. The 12 polygynous colonies sampled were identified when single nests or single nest fragments contained multiple queens, but again we considered all multi-queen colonies to be polygynous, although we were not able to ascertain if all queens within in a nest were reproductively viable or contributing brood. The polygynous nests collected in urban areas, such as south clade samples 18 & 19 and north clade samples 30 & 36, had multiple nest sites along building margins which demonstrated no worker aggression between nests and contained millions of workers and thousands of queens, whereas the polygynous colonies collected from natural areas, such as mountain clade sample 14 and north clade sample 38, were isolated nests under a rock or log with fewer than 20 queens.

### Genetic Data

**Figure 2:**
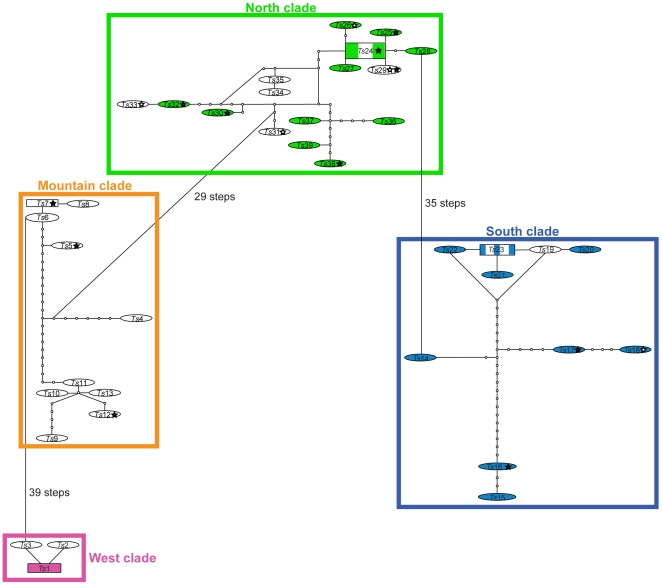
Minimum spanning network calculated by TCS 1.21 using mitochondrial COI sequences. Haplotypes with the highest ancestral probability are displayed as squares, while other haplotypes are displayed as ovals. Shaded boxes/ovals represent urban samples, hollow stars are monogynous colonies, filled stars are polygynous colonies, and colors correspond to Figure 1. Colored boxes define networks identified with 90% probability of parsimony. Connections between networks were determined through decreasing the probability to a minimum of 60%.

A 658-bp fragment of the COI region was amplified and sequenced for 67 specimens of *T. sessile* and a single specimen of *T. erraticum*. Where multiple individuals from a single nest were sequenced a single within-nest haplotype resulted. Overall, there were 39 unique *T. sessile* haplotypes. Of the 658 nucleotide sites in the aligned data matrix and included in the phylogenetic analysis, 115 were variable and 105 were parsimony informative. Haplotypes are named *Ts*-1 to *Ts*-39 and the corresponding GenBank accession numbers are (GU373531-GU373569, GU388394). Average base frequencies were A:0.295, C:0.184, G:0.137, T:0.384. Widespread haplotypes were few, and most clades were comprised of predominantly unique haplotypes (Fig. 2, Appendix S1). No haplotypes were shared between clades (Fig. 2).

### Phylogenetic Analysis

**Figure 3:**
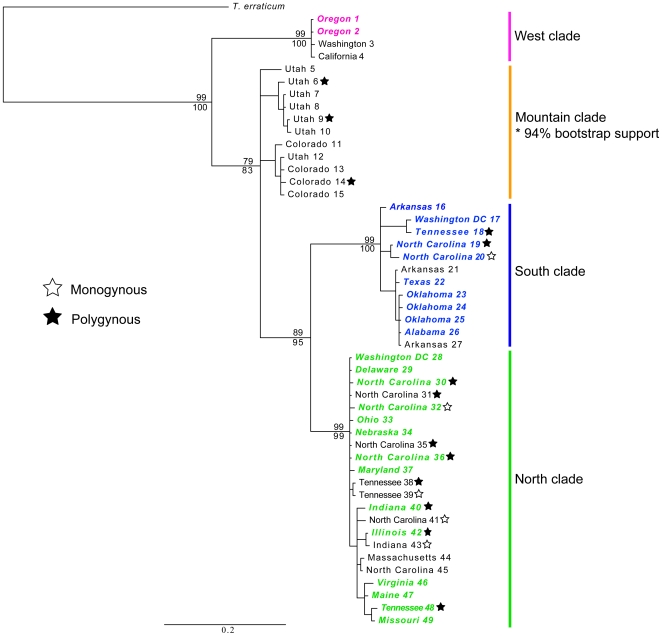
Fifty percent majority rule consensus tree from the Bayesian analysis. Numbers below branches indicate posterior probability values and numbers above indicate nonparametric bootstrap values from the Neighbor-Joining method. Only values >50% (or 0.5 posterior probability) are shown. Colors correspond to Figure 1. Samples denoted with a white star refer to monogynous colonies and black stars refer to polygynous colonies. Taxon labels in color represent urban samples.

Identical consensus tree topologies where produced under the four methods investigated using MEGA, (Bootstrap support for NJ method reported in Fig. 3). MrModeltest identified the GTR+G+I model as best fitting the data. Bayesian analysis produced 9,000 trees after disregarding the burn-in (Fig. 3) and strongly supported the monophyly of a western clade (posterior probability of 100, bootstrap support of 99%). With a posterior probability of 83 and bootstrap support of 79%, a polyphyletic mountain clade was separated from strongly supported monophyletic southern and northern clades (posterior probabilities of 100 and 99, respectively, and bootstrap support of 99% each).

Minimum spanning networks (TCS) analysis of mtDNA sequences produced four unconnected haplotype networks (Fig. 2) with 95% probability of parsimony, corresponding to the four geographic clades identified in figure 3. The west clade consisted of haplotypes *Ts*1 through *Ts*3; the mountain clade haplotypes *Ts*4 through *Ts*13; the south clade haplotypes *Ts*14 through *Ts*23; and the north clade consisted of haplotypes *Ts*24 through *Ts*39. Networks were connected by reducing the probability of parsimony to 60%. In doing this, the north clade was connected to both the south and mountain clades by 35 and 29 mutational steps respectively, while the west clade was connected only to the mountain clade by an additional 39 mutational steps (Fig. 2).

The genetic structure of the different clades was found to be strongly associated with the geographic distribution of the samples. AMOVA results revealed that variation between clades explained 81.5% of the genetic variation (Table 1). Variation within clades also proved significant corresponding to 18.5% of the variation. Kimura-2 parameter pairwise distances between clades ranged from 0.072 to 0.103. Within-clade distances ranged between 0.002 in the western clade and 0.022 in the mountain clade (Table 2). There was an area of broad overlap between the south and north clades along the eastern coast of the United States with samples from Washington DC (17), Knoxville, Tennessee (18), and Rocky Mount and Raleigh in North Carolina (19 and 20) clustering with the south clade, while other samples from Washington DC (28), the mountain regions in Tennessee (38, 39, 48), and numerous other sites in North Carolina (30–32, 35–36, 41, 45) clustered with the north clade (Figs. 1 & 2). Despite this area of broad geographic overlap, the two clades had strong bootstrap and Bayesian support (89% and 95%, respectively) and large differences (7.4%) in average sequence divergence (Fig. 3, Table 2).

**Table 1:** AMOVA results for DNA sequence data analysis of *Tapinoma* group by clade, habitat type, or habitat within north clade.

Source of variation		df	SS	Variance components	Percentage of variation	*p*-Value
**Clade**	Among groups	3	675.77	19.84	81.51	<0.0001
	Among populations within groups	45	202.54	4.5	18.49	<0.0001
**Habitat**	Among groups	1	81.27	2.64	13.45	0.002
	Among populations within groups	47	797.04	16.96	86.55	<0.0001
**Habitat within**	Among groups	1	3.49	0.02	0.55	0.53
**North clade**	Among populations within groups	20	66.11	3.31	99.45	<0.0001

**Table 2:** Average Kimura-2 parameter distances with bootstrap standard errors based on 658 base pairs of the mitochondrial COI gene within a clade on the diagonal in bold and between clades beneath the diagonal.

	North	South	Mountain	West
**North**	**0.011±0.002**			
**South**	0.074±0.009	**0.018±0.003**		
**Mountain**	0.072±0.010	0.081±0.010	**0.022±0.004**	
**West**	0.101±0.012	0.103±0.012	0.083±0.011	**0.002±0.001**

Samples collected from urban environments were not found to be derived from a single source location, but instead were more closely related to geographically proximate samples from natural habitats. AMOVA performed over all samples, regardless of clade, revealed significant variation between samples grouped by habitat (Table 1). This is likely to have resulted from variation existing between clades, and thus not actual differentiation based solely on habitat type. When analysis was performed using samples from a single clade (only the North contained sufficient samples to test significance) no significant variation was detected between groups (Urban vs. Natural). More than 99% of the variation was explained among populations within groups. For example, north clade samples from natural habitats in Indiana (43), North Carolina (31), and Tennessee (38 & 39) clustered with samples from urban sites in Indiana (40), North Carolina (32), and Tennessee (48), respectively (Fig. 3). Similarly, in the south clade samples 21 & 27, collected from forest sites in Arkansas, clustered with samples from urban sites in Alabama (26), Arkansas (16), Oklahoma (23–25), and Texas (22). Single haplotypes were found to contain samples from both urban and natural biomes (*Ts*23 & *Ts*24) (Fig. 2).

Colony structure also varied within each clade in which it was recorded and within different habitats. Polygynous colonies were commonly found in both urban and natural environments and in each of the three clades in which colony type was recorded (Fig. 3, Appendix S1). Monogynous colonies were also collected in both the south and north clades in both urban and natural environments, but no confirmed monogynous colonies were collected in the mountain clade. Monogynous and polygynous colonies were often collected in close proximity to each other, such as samples 38 & 39 and 35 & 41 (Fig. 3, Appendix S1) and samples of both colony structure type were found to share haplotypes (*Ts*29) (Fig. 2).

## Discussion

Our phylogenetic analysis of *T. sessile* collections from the mainland U.S. identified four well supported clades: one found in the western U.S., one in the mountain region (Utah and Colorado), a third spanning the north-central to northeastern U.S., and a fourth covering the south-central to southeastern U.S. (Fig. 1). Some mixing of haplotypes was noted within North Carolina, Washington DC and Tennessee, indicating that this region may act as a break point between the north and south clades (Fig. 1). Within clades, genetic distances were low but significant indicating that within region population genetic structure may exist. The level of genetic difference detected between clades suggests that separate clades may represent unique evolutionary lineages and warrant further investigation (Table 2). It is important to note, however, that our collection sites represented three broadly discontinuous regions (western, mountain, midwestern/eastern), and that further collections between these and other geographic regions could unveil more clades and transition zones between clades.

### Do Urban Colonies Have a Single Common Origin?

Based on evidence of significant genetic variation at the regional level, no differences between habitats, and overlapping haplotypes (Figs. 2 & 3, Table 1), we reject our initial hypothesis that urban populations have become established following a single invasion event with subsequent human-mediated dispersal responsible for a continent-wide distribution. Under this hypothesis, we expected urban samples to form a monophyletic group separate from those derived from natural populations. Our results favor the alternative and to our minds more interesting hypothesis that urban populations of *T. sessile* have arisen multiple times from nearby populations in natural habitats.

### Is Polygynous Colony Structure Associated with the Transition to Urban Habitats?

Based on both behavioral observations and phylogenetic analysis we reject the hypothesis that polygynous colonies are solely associated with urban environments. Rather, colony structure appears to be a plastic trait across habitat type, clade, and haplotype (Figs. 2 & 3). Although the polygynous nests in urban environments had the greatest observed density of both queens and workers compared to nests in natural habitats, colony size varied dramatically within a given environment. For example, within a natural grassland, nests ranged in size from monogynous with 250 workers (39) to polygynous with 5 queens and 960 workers (38), while in urban residential areas both small monogynous (20) and very large polygynous nests with millions of workers and thousands of queens (19) were collected. Our results suggest that multiple lineages of *T. sessile* have been able to successfully invade the urban environment and that this may be due to the plastic expression of colony structure.

### Does *Tapinoma sessile* Have Phylogeographic Structure?

Our results, based on within- and between-clade divergence at the mitochondrial gene COI, suggest that the taxonomic status of *T. sessile* warrants further investigation; it may be comprised of a species complex. In recent years, the taxonomic status of many “species” has been called into question following the application of DNA bar-coding techniques [39], [47]–[50]. Sequence divergence at COI is generally higher between than within species [51], [52]. For example, intraspecific sequence divergence in COI ranged from 0.27% to 1.24% among 256 bird species, while average interspecific sequence divergence was 7.9% for 260 bird species [53]. Similarly, intraspecific sequence divergences ranged from 0–3.2% in the Lycaenidae butterfly family, while the average interspecific divergence was 9.4% [54]. Recent work on ants by Fisher & Smith [55] reported intraspecific variation reaching almost 8% among eight different species of ants in Madagascar, while Wild [51] found intraspecific variation in 11 species of ants in the genus *Linepithema* did not exceed 5%. Therefore, divergence values exceeding the greatest reported intraspecific range (i.e., >8%) in morphologically and molecularly described ant species [55] may therefore indicate the presence of unrecognized sibling species. Moreover, wide ranging species that occupy a diverse array of ecological niches may represent cryptic species complexes. Hebert et al. [39], for example, recommended splitting the species recognized as the neotropical skipper butterfly, *Astraptes fulgerator*, a common and widely distributed species originally described in 1775, into 10 largely sympatric species based on DNA bar-coding at the COI gene along with morphological and natural history information. In the case of *T. sessile*, recent but limited molecular evidence presented in a poster by Smith et al. [56] demonstrated sequence divergence of 9% and preliminary morphological evidence by Hamm [57] together suggest that *T. sessile* may represent multiple cryptic species. Our results, from sampling across the U.S. showing within-clade divergence ranging from 0.2 to 2.3% and among-clade divergence of 7.5 to 10.0%, offer support for the idea that the taxonomic status of *T. sessile* warrants further investigation. It is not surprising that *T. sessile* may represent multiple species given that *T. sessile* occupies the widest geographic range of any native North American ant species and tolerates diverse ecological environments [58]. But, our current sampling does not reveal an obvious correlation with habitat preferences, given that both natural and urban populations were intermixed.

*Tapinoma sessile* is an intriguing case-study because throughout its geographic distribution it occurs in adjacent natural and urban environments (Fig. 1 & 2) and there appears to be a great deal of plasticity in colony structure both within a habitat and between urban and natural environments. How colony structure and queen number are determined in ants has a long history as an evolutionarily important question [59]–[61]. It remains uncertain if the large colonies in urban environments represent a change in colony structure throughout urban habitats, and if so, whether they represent the evolution of a novel phenotype (unicoloniality) or a plastic expansion of existing variation in phenotype of colony structure that might have been favored historically due to the generalist nature of *T. sessile*. A similar situation occurs in the little fire ant (*Wasmannia auropunctata*) in its native range where plasticity in colony type and reproductive structure have been reported along a habitat disturbance gradient [62], [63]. Expansion in colony size among social insects is associated with ecological success, and in ants it has been demonstrated that colonies become more effective competitors when numerically superior [36], [64]. It has also been suggested that more behaviorally dominant ants succeed in urban environments [65]. The suggested importance of behavioral dominance is interesting, given that *T. sessile* in most natural studies has been regarded as an insinuator rather than a dominant species [36], [66]. Nevertheless, we have observed that extremely large colonies of *T. sessile* in urban environments dominate the urban landscape and control baits in the presence of several aggressive invasive ant species, including *Solenopsis invicta* and *Tetramorium caespitum* (personal observation). This apparent expansion in colony size in urban populations of *T. sessile* is similar to the change in colony size among invasive social insects globally [25], [67], [68] and suggests the intriguing possibility that success in urban environments is not unlike succeeding on new continents. If so, both urbanization and species introductions may be favoring a suite of species with similar traits.

A key remaining question with regard to both urban adapted species and invasive species, is how large, typically polygynous colonies arise and what benefits such colonies provide for success in novel environments [25], [60], [67]. A recent review found that unicoloniality and large colonies with multiple queens, more generally, is very common in introduced species [14], [25]. Perhaps the simplest explanation is that such colonies can arise through multiple mechanisms. In some cases, they may result from population bottlenecks like in the Argentine ant, *Linepithema humile* [69], [70], although this does not appear to be the case in *T. sessile*. In other cases, they may result from shifts in the frequency of particular genes, like in the red-imported fire ant, *Solenopsis invicta*, where colony structure is determined by the Gp-9 gene [71]–[73]. Alternatively, they may represent the facultative expression of a phenotype that for whatever reason is successful in new and disturbed environments, which appears to be the case in the little fire ant, *Wasmannia auropunctata*, in both its native and invasive range [62], [63]. This we suspect is the case for *T. sessile*. Surprisingly though, it remains unclear just what advantages such large multiqueen colonies bring ants [59], [60]. It may be that such colonies best take advantage of patchy environments in urban and disturbed habitats, but we know so little that it is difficult even to say if such habitats are more or less patchy from the perspective of ants. While we now know a great deal about some common invasive ant species such as the red-imported fire ant [73], the little fire ant [61]–[63], and Argentine ants [17], [74]–[80], *T. sessile* the most common ant in North America offers many remaining mysteries, from the West coast to the East coast and the deserts nearly all the way to the tundra, this species or group of species succeeds.

## Supporting Information

Appendix S1 List of all colonies used in the study, including the number of workers sequenced, Haplotype, Genbank accession number, and museum where physical specimens are deposited.
(0.03 MB XLS)
